# Yield of Multimodal Imaging in Iris Amelanotic Lesions: A Masked Case-Control Study

**DOI:** 10.3390/diseases13040099

**Published:** 2025-03-28

**Authors:** Rachel Shemesh, Iris Moroz, Meira Neudorfer, Vicktoria Vishnevskia-Dai

**Affiliations:** 1 Faculty of Medical & Health Sciences, Tel Aviv University, Tel Aviv 69978, Israel; shemeshrach@gmail.com (R.S.); iris.moroz@sheba.health.gov.il (I.M.); meiraneu@netvision.net.il (M.N.); 2 Ocular Oncology, Goldschleger Eye Institute, Sheba Medical Center, Ramat-Gan 5262000, Israel; 3 Department of Ophthalmology, Tel Aviv Sourasky Medical Center, Tel Aviv 6423906, Israel; 4 The Chaim Sheba Medical Center, Tel Hashomer 52621, Israel

**Keywords:** iris amelanotic lesions, iris melanoma, iris nevi, multimodal imaging, anterior segment optical coherence tomography (AS-OCT), ultrasound biomicroscopy (UBM)

## Abstract

Objectives: To examine the yield of multimodal imaging of iris amelanotic lesions and evaluate the clinical relevance of these imaging techniques. Methods: In this masked case-control study, imaging, including slit lamp photos, ultrasound biomicroscopy (UBM), and anterior segment optical coherence tomography (AS-OCT) scans of patients diagnosed with iris amelanotic lesions were examined. Seven patients diagnosed with an iris amelanotic lesion were matched by gender and age to seven melanotic iris nevi of similar size and location. Two ocular imaging experts assessed the images in a masked manner and identified which lesion was melanotic and which was amelanotic based on their characteristics. Results: From 2010 to 2021, seven patients were diagnosed with amelanotic iris lesions. All were female; the mean (±SD) age at presentation was 46.3 years (±18). These patients were matched with seven female patients with pigmented iris lesions, with a mean (±SD) age of 49.8 years (±20). Hypopigmented lesions were hyporreflective and had minimal shadowing of the iris behind them, and the basement membrane of the iris could be seen on AS-OCT. Hypopigmented lesions could be delineated from the iris stroma on AS-OCT. In contrast, hyperpigmented lesions were hyperreflective, with significant shadowing blocking the basement membrane and iris stroma. Conclusions: AS-OCT is non-touch and provides substantial information about diagnosing amelanotic nevi. It is more widely available and, in many countries, it is performed by technicians, thus freeing the physician’s time and increasing productivity.

## 1. Introduction

Iris nevus is a common finding that includes both melanocytic (pigmented) and amelanocytic (non-pigmented) lesions [1]. Amelanotic iris nevi are quite rare. Some of the lesions are flat and transparent and therefore can sometimes be overlooked, especially in light-colored irises. When looking at iris amelanotic lesions, iris nevus, iris cyst, hamartomas, metastasis, and melanoma are the most common differential diagnoses [2,3]. Iris cysts classically remain stable as opposed to iris nevi, which can, rarely, grow into melanoma. In addition, hamartomas are usually calcified, and metastases are usually an elevated mass, unlike a flat nevus [2,3]. In an analysis of 3680 iris tumors, iris nevi were the most common lesion, representing 25% of all iris lesions in children, 36% of all iris lesions in young adults, and 47% of all iris lesions in middle-aged and senior adults [4]. In the analysis by Shields et al., 1611 cases of iris nevi referred for evaluation at an ocular oncology center were evaluated. Growth into melanoma occurred in 8% of cases over 15 years. The total number of all hypopigmented lesions, including non-nevi such as hamartomas and vascular lesions, was 358, yet the number of amelanotic nevi and melanoma is not mentioned [4]. Numerous reports of clinical and histopathologic prognostics and outcomes for treatment have been published on iris nevi and melanoma [5,6,7,8]. Krohn et al. studied the clinical features of 29 iris melanomas and found that only one was amelanotic [9]. The risk of iris growth into melanoma is typically clinical, based on features such as ABCDEF: Age young, Blood (hyphema), Clock hour inferior, Diffuse configuration, Ectropion uveae, and Feathery tumor margin, as identified by Shields et al. [10]. However, multimodal imaging techniques, especially anterior segment optical coherence tomography (AS-OCT) and ultrasound biomicroscopy (UBM), improve the accuracy and delimitation of their attributes and extensions [10]. To the best of our knowledge, there is no study that focuses on the imaging features of amelanotic iris nevi and amelanotic melanoma. This study aims to present a small series of iris amelanotic lesions (nevi or melanoma) with imaging characteristics in order to examine the clinical relevance of these imaging techniques and improve the diagnosis of these lesions.

## 2. Materials and Methods

### 2.1. Participants

The inclusion criteria adopted in this study were participants, 18 years of age or older, who had been diagnosed with iris amelanotic nevi and melanoma between 2010 and 2021 at the Sheba Medical Center and had undergone imaging as detailed below. This study excluded metastases, hamartomas, cysts, Lisch nodules, and similar lesions, as it focused on the most common lesion, nevi. The patient diagnosed with melanoma underwent a biopsy and was diagnosed with iris amelanotic melanoma. The lesion had a basal diameter of 2.09 mm and a thickness of 1.52 mm. The histopathological results revealed a spindle cell lesion composed of poorly defined fascicles. The patients with iris amelanotic nevi did not undergo a biopsy, yet follow-up was mandated. Exclusion criteria were applied to help refine the data, and included patients younger than 18 years of age and patients with unclear imaging scans as determined by an ocular imaging expert.

### 2.2. Imaging

Imaging, including slit lamp photos (Topcon BG-4 and DC-4 units), AS-OCT (Heidelberg Engineering Spectralis), and UBM (ABSolu V1.0.4), of patients diagnosed with iris amelanotic nevi and melanoma between 2010 and 2021 at the Sheba Medical Center, were examined. The data were accessed for research purposes on 1 November 2022. Iris amelanotic lesions were matched by gender and age to melanotic iris lesions of similar size and location. Two masked ocular imaging experts received the gender- and age-matched imaging (one of a hypopigmented lesion and one of a pigmented lesion) and determined which lesion is melanotic and which is amelanotic. Following the masked assessment of the UBM and AS-OCT, slit lamp photos were matched to the examination by an additional physician who had access to the patient’s files and pathology if a biopsy was done to examine the accuracy of distinguishing between amelanotic and melanotic lesions based on the UBM and AS-OCT images alone. The research was overseen by the Sheba Medical Center’s formal ethics committee and adhered to the tenets of the Helsinki Declaration (7710-20 SMC). All methods were carried out in accordance with relevant guidelines and regulations. The ethics committee waived informed consent from participants because data were collected retrospectively and anonymously. The study did not involve minors.

## 3. Results

**Figure 1:**
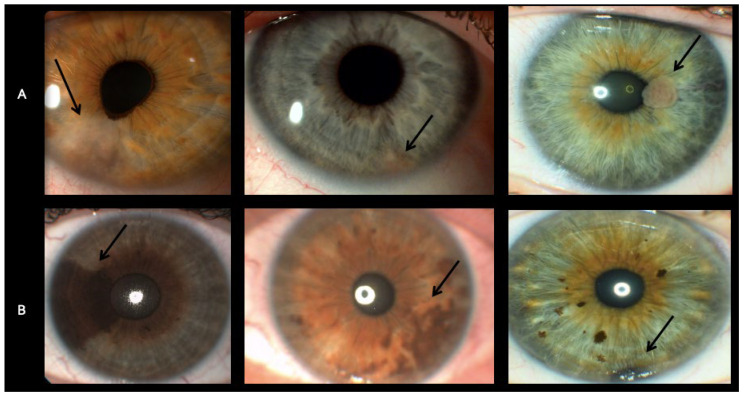
Slit lamp photos (Topcon BG-4 and DC-4 units) of (**A**) iris amelanotic nevi, as previously shown by our group [11]; (**B**) iris melanotic nevi. The arrows in Figure 1 denote the location of the amelanotic lesions.

From 2010 to 2021, seven patients were diagnosed with iris amelanotic lesions (Figure 1A), as was previously shown by our group [11]. All were female; the mean (±SD) age at presentation was 46.3 years (±18), with a range of 22–72 years. These patients’ UBM and AS-OCT scans were matched by gender and age, with a mean (±SD) age of 49.8 years (±20) and a range of 20–69 years, with scans from seven patients diagnosed with iris melanotic nevi (Figure 1B). Patient demographics and clinical characteristics are presented in Table 1.

**Figure 2:**
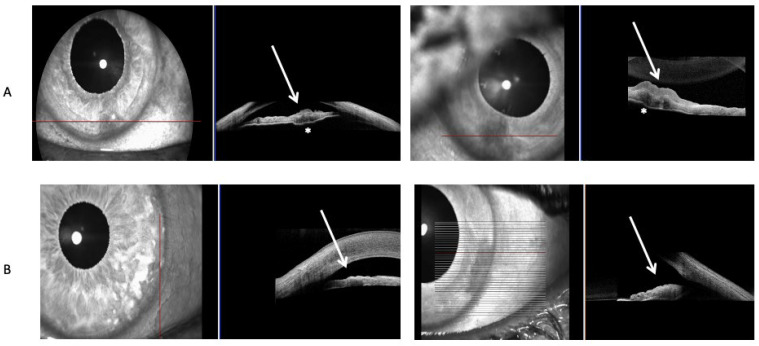
Anterior segment optical coherence tomography scans (Heidelberg Engineering Spectralis) of (**A**) iris amelanotic nevi, demonstrating iris hyporreflective lesions above the iris stroma with minimal shadowing effect behind the iris. The basement membrane of the iris is marked with an asterisk; and (**B**) iris melanotic nevi demonstrating hyperreflective lesions with shadowing below. The basement membrane of the iris is not seen in the scans. The arrows in Figure 2 denote the location of the lesions.

**Figure 3:**
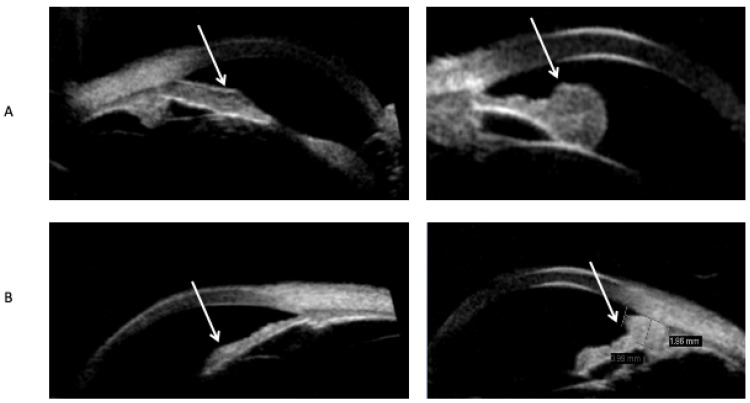
Ultrasound biomicroscopy scans (ABSolu V1.0.4) demonstrate (**A**) iris amelanotic nevi that are hypoechogenic, thickened iris lesions; and (**B**) iris melanotic nevi that are hyperechogenic lesions. The arrows in Figure 3 denote the location of the lesions.

**Figure 4:**
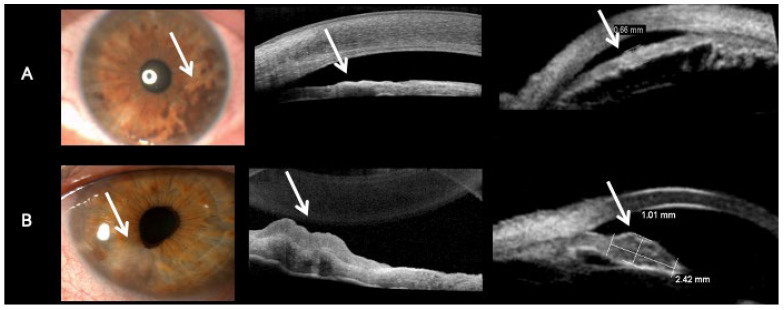
Slit lamp photos (Topcon BG-4 and DC-4 units), anterior segment optical coherence tomography scans (Heidelberg Engineering Spectralis), and ultrasound biomicroscopy scans (ABSolu V1.0.4) demonstrate an (**A**) iris melanotic nevus; and (**B**) iris amelanotic nevus. (**A**) The AS-OCT scan demonstrates a hyperreflective lesion with shadowing below. The basement membrane of the iris is not seen in the scan. The iris melanotic nevus is hyerechogenic on the UBM scan. (**B**) The AS-OCT scan demonstrates a hyporreflective lesion above the iris stroma with minimal shadowing effect behind the iris. The basement membrane of the iris is seen below. The iris amelanotic nevus is hypoechogenic on the UBM scan. The arrows in Figure 4 denote the location of the lesions.

Hypopigmented lesions were hyporreflective and had minimal to no shadowing of the iris behind them, and the basement membrane of the iris could be seen on AS-OCT scans of iris amelanotic nevi (Figure 2A). Hypopigmented lesions could be delineated from the iris stroma on AS-OCT (Figure 2A). In contrast, hyperpigmented lesions were hyperreflective, with significant shadowing blocking the basement membrane and iris stroma (Figure 2B). Hypopigmented lesions on the UBM scan were hypoechogenic (Figure 3A), and hyperpigmented lesions were hyperechogenic (Figure 3B). The multimodal approach included imaging experts in both UBM and AS-OCT imaging, who assessed both scans of matched-pair iris lesions (Figure 4). Out of the seven matched-pair lesions, all lesions were correctly diagnosed as either melanotic or amelanotic lesions by two imaging experts, leading to a diagnostic accuracy of 100 percent in this small case-control study, as assessed by a third physician who examined the file of each patient, including the ocular exam for all patients and the pathology from the biopsy that was done in the iris amelanotic melanoma case. Both UBM and OCT were examined, as AS-OCT enables generally good visualization of the internal structure of the lesion, yet UBM enables a better view of the lesions’ margins and can detect ciliary body involvement.

## 4. Discussion

The current study demonstrates the imaging characteristics for rare amelanotic iris nevi and melanoma. We compared the amelanotic nevi to melanotic nevi, as melanotic nevi are a common finding on ophthalmic exams, and thus the imaging characteristics of these lesions are better known. Two imaging experts were able to distinguish and correctly diagnose melanotic and amelanotic iris nevi and melanoma just by analyzing the UBM and AS-OCT scans. They found that hypopigmented lesions could be delineated from the iris stroma on AS-OCT, as opposed to hyperpigmented lesions that were hyperreflective, with significant shadowing blocking the basement membrane and iris stroma. In addition, hypopigmented lesions on the UBM scan were hypoechogenic, and hyperpigmented lesions were hyperechogenic. This could be useful, as these imaging characteristics can help technicians identify and differentiate these lesions. The limitation is that this assessment does not include a clinical exam of the patient, and thus can miss important information, such as risk factors for lesion growth [10]. However, as this study included a masked evaluation of seven matched cases, the imaging experts did not receive the color images beforehand. This suggests that using AS-OCT can enable the diagnosis of melanotic and amelanotic lesions. As AS-OCT scans are often done by a technician, and UBM is usually done by a professional physician and is not available at every facility, this could mean that if an amelanotic lesion is detected on a clinical exam, then the first imaging approach should be an AS-OCT. If it shows increased thickness, deep changes, or a large mass, we can continue with a UBM scan. The advantages of the different imaging techniques for amelanotic and melanotic lesions are described in Table 2.

Iris amelanotic nevi and melanoma are very rare. In a ten-year cohort of Shemesh et al. [11], 173 patients were diagnosed with iris nevi (melanotic or amelanotic), and the percentage of iris melanotic melanoma was significantly higher than the percentage of iris amelanotic melanoma: 16 (9%) vs. one (0.6%). Rapata et al.’s retrospective study of iris melanocytic tumors in New Zealand over 20 years found 51 tumors, of which only five (10.4%) were amelanotic and 16 were melanomas; yet it is not mentioned how many amelanotic iris melanomas were found [12]. Only one of the 29 (3.4%) iris melanomas studied by Krohn et al. was amelanotic [9]. As many amelanotic lesions are transparent, they may be difficult to diagnose. The purpose of this study was to examine the imaging characteristics of iris amelanotic lesions using multimodal imaging in order to study the yield of these imaging techniques in the diagnosis of amelanotic lesions. Treatment of the underlying malignancy is crucial, hence the importance of a correct and prompt diagnosis aided by multimodal imaging. In this case-control study, we evaluated UBM and AS-OCT scans of iris lesions (nevi and melanoma). Six amelanotic nevi and one amelanotic melanoma were diagnosed at a large tertiary hospital in Israel in the last 10 years. This study demonstrates that iris lesions can be correctly diagnosed as either melanotic or amelanotic lesions based on multimodal imaging, especially with the aid of AS-OCT. Only a few studies have been published on the use of multimodal imaging in the evaluation and diagnosis of iris amelanotic nevi (Table 3). To the best of our knowledge, this is the first study focusing on multimodal imaging of iris amelanotic lesions only. The study of Velazquez-Martin et al. [13] used UBM to analyze ciliary body changes in patients with unilateral oculodermal melanocytosis using the unaffected fellow eyes for comparison, and found increased thickness and reflectivity of the ciliary body due to both augmented melanocyte cells and increased pigmentation. UBM was shown to be helpful in the diagnosis of iris cysts [14] and iris pigmented lesions [15,16]. UBM may help demonstrate hypopigmented lesions as hypoechogenic lesions, as shown in our report and in a previous study [17]. In contrast to melanotic lesions, amelanotic lesions lack melanin [18], allowing the laser beam to pass through the lesion and produce a clear, detailed image. The use of AS-OCT in the diagnosis of iris lesions was demonstrated to be beneficial in the diagnosis of bilateral diffuse uveal melanocytic proliferation (BDUMP) iris lesions [19], nodular iris lesions in anterior uveitis [20,21], diffuse iris juvenile xanthogranuloma [22], and iris arteriovenous malformation (IAVM) [23]. Mirzayev’s recent systematic review on the usage of AS-OCT in the diagnosis of iris nevi has proven that AS-OCT is useful as a noninvasive tool for the diagnosis of solid tumors involving the iris or angle with no marked ciliary body involvement, iris pigment epithelial cysts, and iris stromal cysts [24]. AS-OCT allows for the assessment of the conjunctiva, sclera, cornea, and irido-corneal angle. Although the method provides good imaging of the frontal surface of pigmentation, it has limited ability to penetrate deeper for the assessment of the posterior margins of pigmented lesions and the ciliary body [25]. The study by Wang et al. demonstrated that AS-OCT creates cross-sectional images of the anterior segment angle, iris, and anterior chamber and obtains quantitative parameters in oculodermal melanocytosis and glaucoma related to this condition [26]. Using AS-OCT imaging methods to visualize iris lesion features such as location and thickness, and to determine whether lesions are solid or cystic, limited to the iris, or involve the ciliary body, may facilitate diagnosis and differentiation between benign and malignant iris lesions, thus affecting treatment planning in patients with iris or irido-ciliary lesions [27,28]. Eren et al.’s study demonstrated that amelanotic lesions show reflectivity that is equal to or lower than the iris stroma [27] (Table 3). Our study demonstrates that hypopigmented lesions, which are hyporreflective, have minimal to no shadowing of the iris behind them, and the basement membrane of the iris can be seen on AS-OCT, distinguishing them from iris pigmented lesions (Figure 2). Komatsu et al., Kottaridou et al., and Shields et al. have similarly demonstrated that amelanotic lesions have low to moderate reflectivity, which distinguishes them from highly reflective melanotic lesions [29,30,31] (Table 3). The study by Bianciotto et al. compared UBM and AS-OCT in imaging tumors of the anterior segment of the eye and found that UBM provided better overall tumor visualization and better resolution of the posterior margin, whereas AS-OCT provided better resolution of the anterior margin and better overall resolution of the anterior segment anatomy. Better resolution was found with UBM for pigmented tumors as well as for non-pigmented tumors. However, in this study, no difference was found in tumor resolution between UBM and AS-OCT in iris tumors [25]. In contrast, our study suggests that AS-OCT is useful in amelanotic iris tumors, and it may be superior in the diagnosis of these lesions in comparison to UBM. Pavlin et al. [32] demonstrated similar results to our study. Pavlin et al. [32] also demonstrate that UBM could be superior to AS-OCT in imaging highly pigmented and ciliary body tumors due to its high tissue penetration. To note, the AS-OCT exam is a non-touch tool; it is easy to do and is more comfortable for patients in comparison to the UBM exam. AS-OCT scans are often done by a technician, in contrast to UBM scans, which in many countries are done by physicians, thus freeing the physician’s time and increasing productivity.

## 5. Conclusions

In summary, our study demonstrated the benefit of multimodal imaging in melanotic and amelanotic lesions to determine the diagnosis and characteristics of the lesion. The imaging characteristics of the rare amelanotic iris nevi and melanoma include the ability to delineate it from the iris stroma on AS-OCT and its hypoechogenicity on UBM. Using AS-OCT imaging in the diagnosis of iris amelanotic lesions not only reduces manual labor and frees up the physician’s time but also increases productivity, precision, and efficacy. Thus, this novel imaging method is a valid and promising method to facilitate the early diagnosis and management of potentially sight- and life-threatening lesions. The main limitation of the present case-control study is the rather small sample size. In addition, due to the small sample size, this study provides descriptive statistics only. The strengths include the fact that it includes a masked evaluation of seven matched cases, which adds to the limited available knowledge regarding multimodal imaging of this rare finding. In future studies, we intend to include a larger sample size and examine other lesions, such as metastases, hamartomas, cysts, and Lisch nodules.

## Figures and Tables

**Table 1:** Baseline characteristics of patients, amelanotic and melanotic tumors.

Characteristics	Amelanotic Lesions	Melanotic Lesions
**Gender F (n, %)**	(seven, 100)	(seven, 100)
**Iris color (n, %)**		
Blue	(four, 57.1)	(two, 28.6)
Green	(two, 28.6)	(four, 57.1)
Hazel	(one,14.3)	(one, 14.3)
**Age (y)**		
Mean ± SD,	46.3 ± 18.0,	49.8 ± 20.0,
Range [Min, Max]	[22, 72]	[20, 69]
**Eye OD (n, %)**	(five, 71.4)	(two, 28.6)
**Lesion location (n, %)**		
Superior	(one, 14.0)	(three, 42.9)
Inferior	(six, 86.0)	(four, 57.1)
**Lesion size (mm)**		
Tumor diameter	2.1 ± 0.2,	1.9 ± 0.7,
Tumor thickness	0.9 ± 0.4	1.12 ± 0.8
Mean ± SD		
**Symptoms (n, %)**		
Incidental finding	(four, 57.1)	(five, 71.4)
Itching	(two, 28.6)	(one, 14.3)
Vision deterioration	(one, 14.3)	(one, 14.3)
**Pathology (n, %)**		
Iris nevus	(six, 86.0)	(seven, 100.0)
Iris melanoma	(one, 14.0)	(zero, 0.0)

F—female; n—number; SD—standard deviation; min—minimum; max-maximum; y—years, mm—millimeters). The amelanotic lesion demographic data was previously demonstrated by our group [11].

**Table 2:** Comparison of AS-OCT and UBM imaging advantages for amelanotic and melanotic lesions.

Lesion Type	Advantages of AS-OCT Imaging	Advantages of UBM Imaging
Amelanotic Lesions	High-resolution cross-sectional imaging; non-contact, faster procedure; better visualization of superficial structures.	Superior penetration through opaque media (e.g., dense corneal scars); provides better visualization of deeper structures, including ciliary body involvement; effective in assessing lesion thickness and internal reflectivity.
Melanotic Lesions	Can detect hyperreflective pigmentation and surface irregularities; allows differentiation from non-pigmented lesions.	Less light attenuation by pigmented tissues compared to AS-OCT, providing clearer structural details; can assess posterior extension and involvement of deeper structures like the sclera and ciliary body.

**Table 3:** A summary of studies on the imaging findings of amelanotic iris lesions.

Name of Paper	Authors	Year	Ref	AS-OCT Imaging Findings	UBM Imaging Findings
Evaluation of Iris Melanoma with Anterior Segment Optical Coherence Tomography	Eren et al.	2018	[27]	Amelanotic lesions showed reflectivity equal to or lower than iris stroma; heterogeneous internal structure.	Low-to-moderate reflectivity, independent of pigmentation; clear posterior margin visualization.
Imaging of Anterior Segment Tumors: A Comparison of Ultrasound Biomicroscopy Versus Anterior Segment Optical Coherence Tomography	Kottaridou et al.	2024	[29]	Amelanotic lesions depicted with low to moderate reflectivity, distinct from highly reflective melanotic lesions.	Better visualization of all tumour margins and posterior surfaces; complete penetration of lesions.
A Pilot Study to Evaluate the Usefulness of Optical Coherence Tomography for Staging Iris Pigmented Lesions in Cats (extrapolated for human relevance)	Komatsu et al.	2024	[30]	Amelanotic lesions inferred to lack hyperreflective lines seen in pigmented lesions; thinner iris profile.	Not detailed for amelanotic specifically; generally high-resolution imaging of anterior segment.
Iris Melanoma: Features and Prognosis in 317 Children and Adults	Shields et al.	2012	[31]	Amelanotic lesions less reflective than pigmented; structural details visible but not specific to amelanotic lesions.	Low-to-moderate reflectivity; complete penetration showing lesion extent and ciliary body involvement.

AS-OCT—anterior segment optical coherence tomography; UBM—ultrasound biomicroscopy.

## Data Availability

Data available on reasonable request.
